# Properties of An Oral Nanoformulation of A Molecularly Dispersed Amphotericin B Comprising A Composite Matrix of Theobroma Oil and Bee’S Wax

**DOI:** 10.3390/nano4040905

**Published:** 2014-12-19

**Authors:** Chloe See Wei Tan, Nashiru Billa, Clive J. Roberts, David J. Scurr

**Affiliations:** 1 School of Pharmacy, The University of Nottingham, Malaysia Campus, Jalan Broga, Semenyih 43500, Selangor Darul Ehsan, Malaysia; E-Mail: tanseewei@hotmail.co.uk; 2 School of Pharmacy, The University of Nottingham, Park Campus, Nottingham NG7 2RD, UK; E-Mails: clive.roberts@nottingham.ac.uk (C.J.R.); david.scurr@nottingham.ac.uk (D.J.S.)

**Keywords:** amphotericin B (AmB), nanoparticles, theobroma oil, beeswax, lipid, cellular uptake

## Abstract

An amphotericin B-containing (AmB) solid lipid nanoparticulate drug delivery system intended for oral administration, comprised of bee’s wax and theobroma oil as lipid components was formulated with the aim to ascertain the location of AmB within the lipid matrix: (a) a homogenous matrix; (b) a drug-enriched shell; or (c) a drug enriched core. Both the drug-loaded and drug-free nanoparticles were spherical with AmB contributing to an increase in both the *z*-average diameter (169 ± 1 to 222 ± 2 nm) and zeta potential (40.8 ± 0.9 to 50.3 ± 1.0 mV) of the nanoparticles. A maximum encapsulation efficiency of 21.4% ± 3.0%, corresponding to 10.7 ± 0.4 mg encapsulated AmB within the lipid matrix was observed. Surface analysis and electron microscopic imaging indicated that AmB was dispersed uniformly within the lipid matrix (option (a) above) and, therefore, this is the most suitable of the three models with regard to modeling the propensity for uptake by epithelia and release of AmB in lymph.

## 1. Introduction

Invasive fungal infections have been recognized as a major cause of morbidity and mortality among immuno-deficient patients (e.g., transplants, chemotherapy, Acquired Immune Deficiency Syndrome (AIDS)) worldwide. The polyene macrolide, amphotericin B (AmB), which has been in use for over 30 years remains as the drug of choice for managing the above conditions since it is the most effective treatment against several systemic and/or visceral bacterial and fungal infections [1,2,3]. However, AmB is currently administered by injection because it has a low bioavailability via the preferred oral administration route. Oral administration of dosage forms has several advantages, for example, it is the most common and “natural” means of getting medicines into our body and this attribute augments patient compliance. The oral route of delivery is also free from some of the side effects associated with more invasive routes of drug administration. In the case of AmB, nephrotoxicity following parenteral administration has been partly linked to the excipients required in such an injectable formulation [4]. Therefore, research has intensified in the development of orally administered AmB formulations. A key requirement for such delivery systems is that they should be able to match the challenges posed by the gastrointestinal tract, such as stability and absorption issues [4]. The absorption of particles from the gastrointestinal tract has been shown to be influenced by size, lipophilicity and surface characteristics [5]. The presence of lipid moieties on the surface of nanoparticles has been shown to prolong the gastrointestinal residence time of the particles through interaction of the lipids with epithelial membranes, which could lead to enhanced absorption [6,7,8]. The digestion of triglycerides by pancreatic lipase produces surface-active mono and diacylglycerols, which induce secretion of bile salts and the formation of mixed-micelles which facilitate absorption [9,10,11,12]. Furthermore, solid lipid nanoparticles can enhance the lymphatic delivery of drugs which also contributes to bioavailability [10].

Depending on the relative dominance of each of these characteristics within the carrier system, particles can be favorably or unfavorably taken up by the Peyer’s patches of the gastrointestinal epithelia. For instance positively charged NPs are better taken up by pinocytosis compared to negatively charged particles. Particles of sizes below 200 nm are also better taken up [11]. Therefore, it is rational to investigate the location of the payload relative to the carrier system since this information will shed light on the propensity of the particles to be taken up. Once taken up, the particles become assimilated in the lymph and how rapidly the drug is released into the lymph depends in part on the location and state of the drug within the nanoparticles. There are essentially three possible scenarios of drug localization within micron and submicron drug delivery systems as proposed by Muller *et al.* [5] and Mehnert and Mader [13]: (a) a homogenous matrix; (b) a drug-enriched shell; and (c) a drug enriched core.

In the present study, we used a composite of bee’s wax and theobroma oil to formulate an AmB-containing solid lipid nanoparticlate delivery system. These two lipids are considered safe and widely used in the food industry. Our main aim was therefore to ascertain the location of AmB within the solid lipid nanoparticles. This property is important in ascertaining the propensity of the nanoparticle to be taken up by Peyer’s patches and is crucial to the rate of assimilation and release of the drug in lymph. Although discussed in the literature, little has been published on methods that can be used to locate drug distribution within solid lipid nanoparticulate drug delivery systems.

## 2. Experimental Section

### 2.1. Materials

Beeswax, sodium cholate (SC), lecithin and amphotericin B (AmB) were purchased from Sigma (Sigma-Aldrich Co. LLC., St. Louis, MO, USA) whilst Theobroma oil (TO) was obtained from Kondima (Kondima Engelhardt GmbH and Co. KG, Karlsruhe Stösserstraße, Germany). Methanol, chloroform, ethyl acetate and all other reagents were analytical grade and purchased from R&M (Reichle and De-Massari AG, Wetzikon, Switzerland).

### 2.2. Preparation of Lipid Nanoparticles

AmB-containing lipid nanoparticles were formulated by solvent diffusion [14] according to the formula presented in Table 1. Briefly, AmB was first dissolved in a chloroform, along with the lipids (beeswax (BW), oleic acid (OA) and theobroma oil (TO)) and lecithin, followed by evaporation of the organic solvents. The lipid matrix containing the AmB was then dissolved in ethyl acetate at 70 °C. At the same time, 20 mL of the 5% aqueous solution of sodium cholate was heated to the same temperature. Both phases were mixed and then homogenized using a high speed homogenizer (Ika-Turrax) at 10,000 rpm for 10 min. Then, 80 mL of water at 70 °C was added slowly into the mixture with continuous stirring for a further 20 min before being subjected to ultrasonication for 20 min. Finally, the organic solvent was evaporated off.

The weight of AmB were varied (10, 35, 50 and 65 mg) in order to study the effect of amount of AmB loading on the encapsulation efficiency of the method. AmB-free lipid nanoparticles (referred to here as drug-free lipid nanoparticles) were similarly prepared with the omission of AmB.

**Table 1:** Formula of lipid nanoparticles as a function of amount of AmB.

AmB (mg)	20 mL of aqueous sodium cholate solution (% *w*/*w*)	Lecithin (% *w*/*w* of total lipid)	Lipid matrix (mg)		Water (mL)
			Theobroma oil	Beeswax	
10.0; 35.0; 50.0; 65.0	5.0	30	200	200	80

### 2.3. Photon Correlation Spectroscopy Analysis

Photon correlation spectroscopy studies were carried on the nanoparticles using a Zetasizer Nano ZS (Malvern, UK) equipped with a 4 mW He-Ne laser (633 nm). The parameters measured were polydispersity index (PDI), *z*-average diameter and the zeta potential (ξ). Particle size analysis was evaluated using intensity distribution and hence the *z*-average diameter is an intensity mean diameter and the PDI describes the width of the particle size distribution. ξ, which is the surface charge on the nanoparticles was analyzed by the software based on the Henry equation, which takes into account the electrophoretic mobility of the particles. Prior to analysis, each sample was diluted to 0.1% (*w*/*v*) with deionized (DI) water. Each analysis was carried out at 25 °C and performed in triplicate whilst the data are expressed as mean ± standard deviation.

### 2.4. Encapsulation Efficiency

The encapsulation of AmB within the lipid nanoparticles was estimated using high pressure liquid chromatography (HPLC) as described by Tan and Billa [15]. Briefly, a few drops of 0.1 M hydrochloric acid (HCl) was added to a 1 mL aliquot of the prepared formulation in order to precipitate the lipid nanoparticles followed by centrifugation at 11,000 rpm for 45 min at 16 °C. The supernatant was then decanted and 425 µL of DMSO and 100 µL of 0.01% 1-amino-4-nitronaphthalene (internal standard) were added to the pellet and then heated to 70 °C. A 420 µL aliquot of this solution was added to 750 µL of DI water. The amount of AmB was then determined using an HPLC system (PerkinElmer, Shelton, CT, USA) equipped with a 15 cm × 4.6 mm reversed-phase C-18 (Apex ODS, Grace USA, Williamsburg, MI, USA), 5 µm particle size stationary phase. Detection was by ultraviolet at 405 nm. The mobile phase comprised of 70% 2.5 mM EDTA and 30% acetonitrile. Analysis were performed in triplicates and expressed as mean values ± standard deviation. AmB content was determined by calculating the peak-height ratio of AmB to the internal standard and comparing these to the same obtained from the standard curve.

The encapsulation efficiency of AmB within the lipid nanoparticles expressed as percentage (%EE) was calculated using the following relationship:

(1) $$\%\text{EE}=\frac{\text{Amount of drug in precipitate}}{\text{Amount of drug added}}\times 100$$

The amount of drug in precipitate (amount of encapsulated drug) was obtained by multiplying the amount of AmB detected by HPLC with the dilution factor from the sample preparation step where DMSO, 1-amino-4-nitronaphthalene and DI water were added. Each analysis was performed in triplicates and the data are expressed as mean ± standard deviation.

### 2.5. Nanoparticle Tracking Analysis (NTA)

The NTA measurements were performed on a NanoSight LM20 (NanoSight, Amesbury, UK), fitted with a sample chamber and a 640-nm laser emitter. The samples were injected into the chamber using sterile syringes to fill the tip of the nozzle prior to analysis. Analysis of the data were performed using NTA 2.0 Build 125 software which is based on identifying and tracking individual nanoparticles moving under Brownian within the chamber. In this regard, the movement of a particle is related to its size as indicated by the Stokes-Einstein equation [16]:

(2) $$\bar{{\left(x,y\right)}^{2}}=\frac{2k_{B}T}{3r_{h}\pi \eta }$$

where, k*_B_* is the Boltzmann constant and $\bar{{\left(x,y\right)}^{2}}$ is the mean squared speed of a particle at a temperature T, in a medium of viscosity η, with a hydrodynamic radius of r*_h_*. Videos of 100 s were captured at 30 frames per second and analyzed using the NTA software.

### 2.6. Field Emission Scanning Electron Microscopy (FESEM) and Scanning Transmission Electron Microscopy (STEM) Analysis

The surface topography and morphology of the lipid nanoparticles were examined using a Carl Zeiss Supra 55VP-30-86 FE-SEM in high vacuum mode. Prior to analysis, samples were diluted with DI water, air-dried and mounted on the stage. They were then osmium tetraoxide-fixed before viewing under field emission at 4.0 kV. Samples for STEM imaging were similarly prepared without fixation and observed under scanning transmission at 30 kV, using a Carl Zeiss Supra 40VP-31-31 SEM.

### 2.7. Time-of-Flight Secondary Ion Mass Spectrometry (ToF-SIMS) Analysis

The surface functionality of the air-dried nanoparticles was carried out using a secondary ion mass spectrometer (ION-ToF IV, GmbH, Münster, Germany) equipped with a liquid metal bismuth (Bi^+^) ion gun (LMIG). Prior to analysis, AmB-loaded lipid nanoparticles were washed in order to remove any unassociated traces of AmB. The washing was performed by ultracentrifugation (Jouan GR 2022, St. Herblain, France) for 25 min at 16,000 rpm at room temperature with phosphate buffer at pH 7. This procedure was repeated several times until no residue of AmB was detected in the washing buffer when analyzed spectrophotometrically at a 408 nm. The pelletized lipid nanoparticles were re-dispersed in DI water by vortex-mixing and then air-dried before analysis. The mass spectrometer was operated at a pressure of 10^−9^ mbar and a pulse of 25 keV generated from a Bi^+^ primary ion source that delivered a current of 1.1 pA over a 100 µm × 100 µm area. A compact of the dried SLN, or raw material measuring approximately 1 cm × 1 cm was formed within a plastic die so that secondary ions originating from the sample following bombardment by primary ions represent fragments from molecules located on surface of material. The bombardment of primary ions caused secondary particles from the surface of the nanoparticles, *i.e.*, electrons, photons, atoms, and neutral molecules to be emitted. These secondary particles were captured (parallel acquisition with a time-of-flight analyzer) and processing analysis performed using the Ion-Spec software (Version 7.0, ON-ToF GmbH, Münster, Germany). Both positive and negative ion profiles were recorded in duplicate using the same sputtering and analysis conditions.

### 2.8. Statistical Analysis

Statistical analysis were performed using PASW Statistic software version 18.0. The physical properties between formulations were compared when appropriate, using a one-way analysis of variance procedure together with Tukey’s *post hoc* test. In all cases, statistical significant difference was indicated when *p* ≤ 0.01.

## 3. Results and Discussion

### 3.1. Encapsulation Efficiency (%EE)

**Figure 1:**
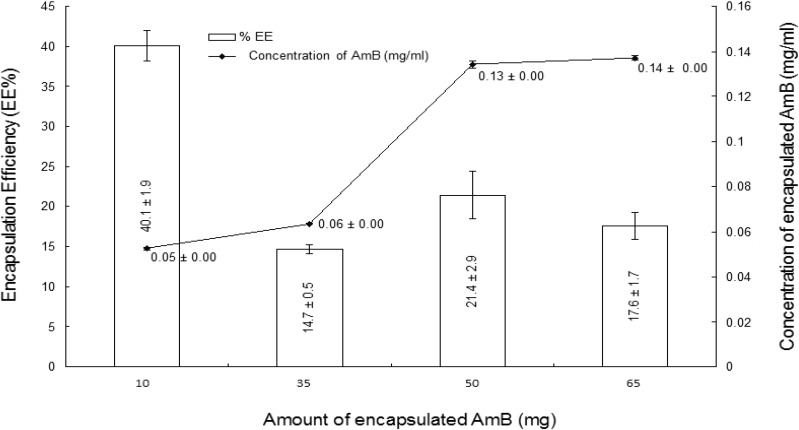
Concentrations and encapsulation efficiencies of AmB within the solid lipid nanoparticles (*n* = 3).

The amount of drug within the carrier nanoparticles is crucial since it relates to the functionality, cost-effectiveness of the method of manufacture and ultimate bioavailability from the delivery system. In the present study, we varied the amounts of AmB (10, 35, 50 and 65 mg) used during formulation at a fixed weight of total lipid to give ratios of 0.025, 0.0875, 0.125, and 0.1625, respectively. The %EE of AmB was > 40% at a ratio of 0.025 AmB to combined weights of lipids (Figure 1) and decreased somewhat as the ratio of AmB increased. This phenomenon has been attributed to the limited space available for localisation at high drug loading and has been observed in lipid matrices loaded with verapamil [17]. Although there was a reduction in %EE with the amount of AmB in the formulation, the actual amount of AmB increased and reached a plateau at a ratio of 0.125. A similar observation has been reported by Hong Yuan *et al.* [18] who showed that there was a reduction in %EE but an improved total loading of progesterone within nanostructured lipid crystals (NLC) with increased progesterone concentration. Hence, a drug to lipid ratio of 0.125 was considered as the optimal. Nanoparticles from this formula yielded a %EE of 21.4% ± 3.0%, corresponding to 10.7 ± 0.4 mg of encapsulated AmB within the lipid matrix. Further characterizations on this formulation were carried out in comparison with the drug-free nanoparticles.

### 3.2. PCS Analysis

There was a significant increase (*p* ≤ 0.01) in both PDI (0.255 ± 0.006) and *z*-average diameter (222 ± 2 nm) in the AmB-containing nanoparticles as shown in Table 2 and as observed elsewhere [18,19,20]. This increase in the above parameters is attributable to an increase in viscosity of the molten lipid phase during formulation when the amount of AmB was increased [18,21]. As the viscosity increased and at a fixed input of shear forces, the molten lipid phase was less efficiently dispersed during homogenisation into smaller droplets, which ultimately manifested as an increase in PDI and *z*-average diameter.

The zeta potential (ξ) of the AmB-loaded lipid nanoparticle was −50.27 ± 1.01 mV being significantly different from that obtained from the drug-free nanoparticles (−40.80 ± 0.9 mV), (*p* ≤ 0.05). Therefore, the presence of AmB caused an increase the surface charge on the lipid nanoparticles. It is documented that the ξ of lipid nanoparticles decreases with an increase in the *z*-average diameter increased and *vice versa* [18,21]. However, this was not observed in the present study, where an increase in the *z*-average diameter was coupled with an increase in ξ of AmB-loaded nanoparticles (*p* ≤ 0.05). This observation points to a contribution to the ξ by AmB which compensates any decrease in the charge density brought about by an increase in *z*-average diameter.

**Table 2:** *Z*-average diameters; polydispersity indices (PDI); zeta potentials (ξ) of drug-free and AmB-loaded lipid nanoparticulate dispersions (*n* = 3).

Formulation	*z*-average diameter (nm)	PDI	ξ (mV)
Drug-free nanoparticles	169 ± 1	0.215 ± 0.023	40.8 ± 0.9
AmB-loaded nanoparticle	222 ± 2	0.255 ± 0.006	50.3 ± 1.0

### 3.3. NTA Analysis

The PCS analysis revealed that the *z*-average diameters of both the drug-free and AmB-containing nanoparticles were in the nanometer range. The PDI values for both were also low and indicated a narrow size distribution profile.

**Figure 2:**
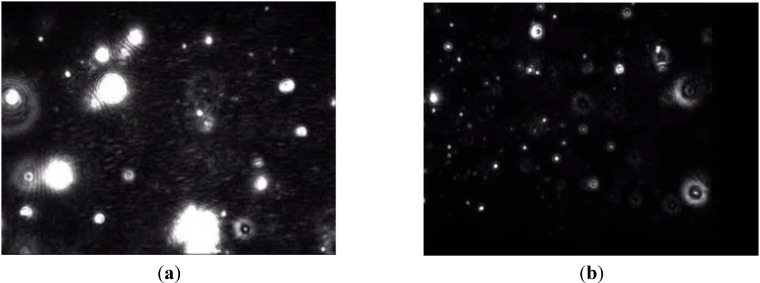
NTA static video images of (**a**) drug-free; (**b**) AmB-loaded solid lipid nanoparticles.

**Figure 3:**
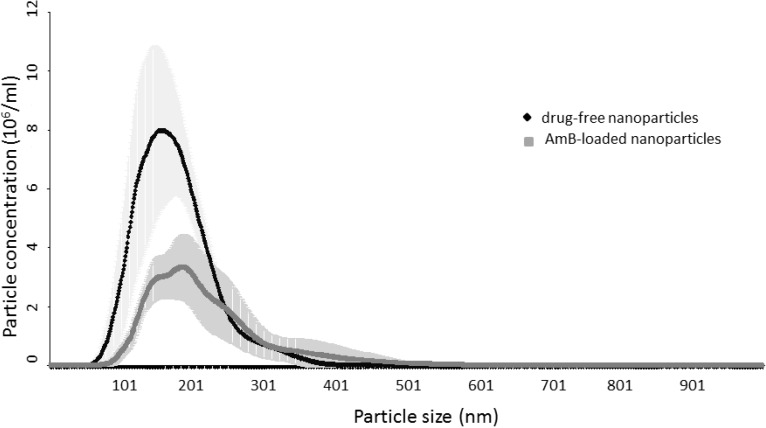
Size distribution profiles from NTA for drug-free and AmB-loaded solid lipid nanoparticles (*n* = 3).

The size distribution data obtained from the NTA analysis show peaks at 155 ± 17 nm and 184 ± 10 nm for drug-free and AmB-loaded lipid nanoparticles respectively. Furthermore, the difference in particle sizes and scattering intensities of these two preparations visualized by NTA revealed that the AmB-loaded lipid nanoparticles were larger as they showed a slower rate of Brownian motion compared to the drug-free nanoparticles (Figure 2). The D10, D50 and D90 (particle diameter corresponding to 10%, 50% and 90% of cumulative undersize particle size distribution, respectively) for the drug-free lipid nanoparticles were 111 ± 9 nm, 165 ± 9 nm, and 243 ± 12 nm, respectively, generating a mean particle size of 173.67 ± 9.07 nm. In contrast, the AmB-loaded lipid nanoparticles had larger corresponding values (D10: 133 ± 10 nm; D50: 196 ± 18 nm, D90: 309 ± 53 nm) and a mean particle size value of 211 ± 23 nm. AmB-loaded lipid nanoparticles also registered a higher degree of size distribution compared to the drug-free nanoparticles as shown in Figure 3 (73 ± 12 for AmB-loaded lipid nanoparticle and 55 ± 2 for drug-free). The results from the NTA study, thus, corroborates with the data obtained from the PCS analysis.

### 3.4. FESEM and STEM Analysis

**Figure 4:**
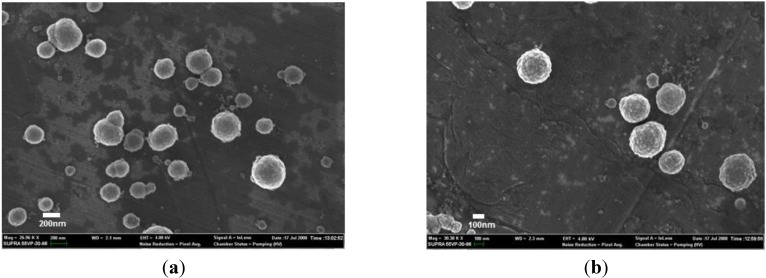
FESEM images of (**a**) drug-free; and (**b**) AmB-loaded solid lipid nanoparticles.

**Figure 5:**
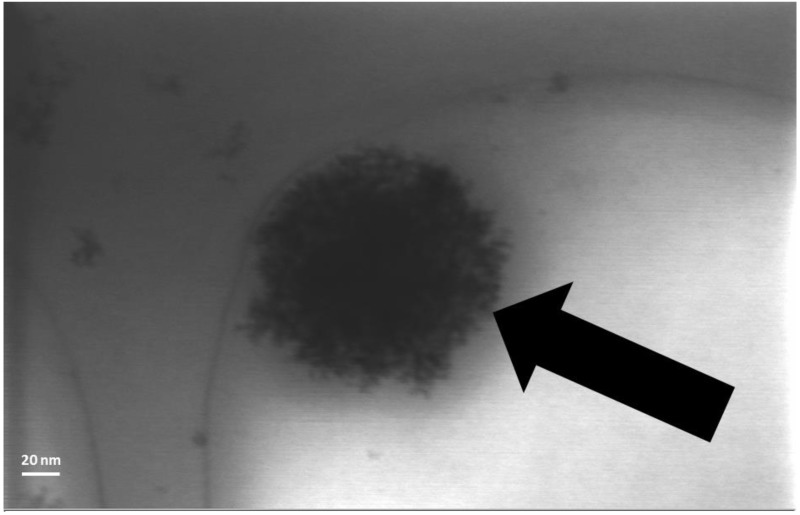
STEM image of an AmB-loaded solid lipid nanoparticle revealing a homogenous internal matrix.

The dimensions of the lipid nanoparticles observed from the PCS analysis agree with those observed from the NTA data and suggest that the AmB loaded and drug-free nanoparticles exist as fairly discrete dispersions. The FESEM analysis indicates that the size range of the particles falls within 250–300 nm for drug-free (Figure 4a) and 300–350 nm for AmB-loaded lipid nanoparticles (Figure 4b). These would seem to be slightly larger than the data obtained from the PCS and NTA analysis. Osmium fixation as used in the sample preparation is known to increase the size of objects due to the coating [16]. The FESEM indicate that the morphologies of both the AmB-containing and drug-free nanoparticles were spherical with a slight degree of agglomeration in the drug-free compared to the AmB-containing nanoparticles. This relative degree in discreteness between the two types of formulations can be attributed to the higher value of ξ within the AmB nanoparticles, which serves to impose better charge repulsions between the nanoparticles. The surfaces of the AmB-containing nanoparticles appear to be rougher compared to the drug-free nanoparticles. The STEM image (Figure 5) of the AmB nanoparticles reveals a uniform matrix, suggesting that AmB is uniformly dispersed within the lipid matrix of the nanoparticles (model a).

### 3.5. Time of Flight Secondary Ion Mass Spectroscopy (ToF-SIMS) Analysis

**Figure 6:**
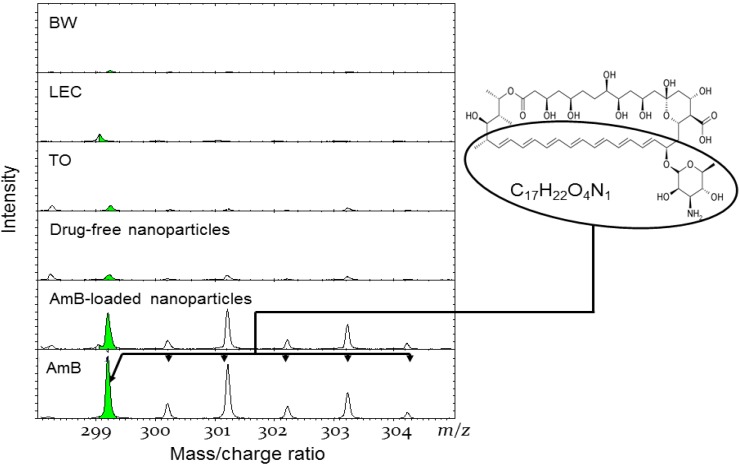
Spectra (m/z) of secondary negative ions obtained from raw material (BW, LEC, AmB and TO) drug-free and AmB-loaded solid lipid nanoparticles.

ToF-SIMS analysis was carried out on nanoparticles and raw materials in order to ascertain which of the three possible models: (a) the homogenous matrix; (b) the drug-enriched shell; or (c) the drug enriched core can be ascribed to the localization of Amb within the nanoparticles. The analysis was based on detection of secondary ion signals from the surfaces of the particles when these surfaces were bombarded with primary ions (Bi^+^) and the potential molecular fragments from the ingredients used in the formulations (BW, LEC, TO, AmB) were captured. The m/z ratio due to pure AmB was observed in the negative spectra at molecular mass ranges of 299.2 to 304.2 and points to a contribution by the [C_17_H_17_O_4_N] moiety of AmB (Figure 6). Crucially, the peak observed at 299.2, with an increment of mass of 1 corresponds to the sputter of one ionized hydrogen from the surface, which gives rise to molecular masses of 300.2, 301.2, 302.2, 303.2, and 304.2. These peaks are clearly indicated in the AmB spectra and the AmB-containing nanoparticles spectra, albeit at lower intensities. The above peaks (299.2, 300.2, 301.2, 302.2, 303.2, and 304.2 m/z) are also faintly noticeable in the spectra of the raw ingredients (TO, BW, and LEC) and the drug-free lipid nanoparticles. Hence, the presence of these peaks within the drug-free nanoparticles spectra was contributed by moieties from the raw ingredients rather than from AmB moieties. We may, thus, conclude that some AmB is present on the surface of the AmB-containing nanoparticles but the fact that the intensities of these peaks are of a lower magnitude compared to pure AmB suggests that the surface of the AmB-containing nanoparticles contains constituents other than AmB and presumably, the lipid composite. Along with the FESEM and STEM analysis, there is a strong indication that AmB is dispersed homogenously within the lipid matrix (model a).

## 4. Conclusions

Theobroma oil and bees wax were successfully used to formulate lipid nanoparticles containing the antifungal AmB. Complementary analysis using a range of techniques indicate that AmB was uniformly dispersed within the lipid matrix and that this caused an increase in the surface charge of the nanoparticles thus that these presented a more discrete dispersion compared to the drug-free nanoparticles. This localization scheme of AmB within the nanoparticles is likely to be favorable in the present context since it is indicative of a possible delayed gastric residence time, combined with a slow release of AmB when delivered orally.
